# (*E*)-1-(5-Bromo­thio­phen-2-yl)-3-(3,4,5-trimeth­oxy­phen­yl)prop-2-en-1-one

**DOI:** 10.1107/S1600536811049294

**Published:** 2011-11-30

**Authors:** Suresh B. Vepuri, H. C. Devarajegowda, Waleed Fadl Ali Al-eryani, K. Lavanya, S. Anbazhagan

**Affiliations:** a Institute of Pharmacy, GITAM University, Visakhapatnam-45, Andhrapradesh, India; b Department of Physics, Yuvaraja’s College (Constituent College), University of Mysore, Mysore 570 005, Karnataka, India; c Karuna College of Pharmacy, Thirumittacode, Palakad 679 533, Kerala, India

## Abstract

In the title compound, C_16_H_15_BrO_4_S, the dihedral angle between the thio­phene and benzene rings is 13.08 (16)°. The C atoms of the *meta* meth­oxy groups of the substituted benzene ring lie close to the plane of the ring [displacements = 0.049 (5) and −0.022 (4) Å], whereas the *para*-C atom is significantly displaced [−1.052 (4) Å]. In the crystal, mol­ecules are linked by weak C—H⋯O hydrogen bonds, forming *C*(11) chains propagating in [100].

## Related literature

For general background to chalcones see: Chun *et al.* (2001 ▶); Horng *et al.* (2003 ▶); Mei *et al.* (2003 ▶).
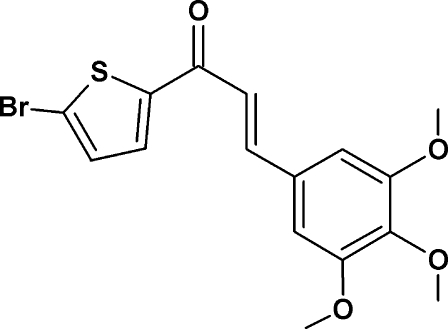


## Experimental

### 

#### Crystal data

C_16_H_15_BrO_4_S
*M* *_r_* = 383.25
Orthorhombic, 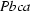

*a* = 16.8923 (7) Å
*b* = 8.0793 (6) Å
*c* = 23.6427 (17) Å
*V* = 3226.7 (4) Å^3^
*Z* = 8
Mo *K*α radiation
μ = 2.69 mm^−1^
*T* = 293 K
0.22 × 0.15 × 0.12 mm

#### Data collection

Oxford Diffraction Xcalibur diffractometer
Absorption correction: multi-scan (*CrysAlis PRO RED*; Oxford Diffraction, 2010 ▶) *T*_min_ = 0.625, *T*_max_ = 1.000
17608 measured reflections
2833 independent reflections
1944 reflections with *I* > 2σ(*I*)
*R*_int_ = 0.055

#### Refinement

*R*[*F* ^2^ > 2σ(*F* ^2^)] = 0.042
*wR*(*F* ^2^) = 0.074
*S* = 0.99
2833 reflections
200 parameters
H-atom parameters constrained
Δρ_max_ = 0.37 e Å^−3^
Δρ_min_ = −0.30 e Å^−3^

### 

Data collection: *CrysAlis PRO CCD* (Oxford Diffraction, 2010 ▶); cell refinement: *CrysAlis PRO CCD*; data reduction: *CrysAlis PRO RED* (Oxford Diffraction, 2010 ▶); program(s) used to solve structure: *SHELXS97* (Sheldrick, 2008 ▶); program(s) used to refine structure: *SHELXL97* (Sheldrick, 2008 ▶); molecular graphics: *ORTEP-3* (Farrugia, 1997 ▶) and *CAMERON* (Watkin *et al.*, 1993 ▶); software used to prepare material for publication: *WinGX* (Farrugia, 1999 ▶).

## Supplementary Material

Crystal structure: contains datablock(s) I, global. DOI: 10.1107/S1600536811049294/hb6511sup1.cif

Structure factors: contains datablock(s) I. DOI: 10.1107/S1600536811049294/hb6511Isup2.hkl

Supplementary material file. DOI: 10.1107/S1600536811049294/hb6511Isup3.cml

Additional supplementary materials:  crystallographic information; 3D view; checkCIF report

## Figures and Tables

**Table 1:** Hydrogen-bond geometry (Å, °)

*D*—H⋯*A*	*D*—H	H⋯*A*	*D*⋯*A*	*D*—H⋯*A*
C21—H21⋯O6^i^	0.93	2.46	3.320 (4)	155

Symmetry code: (i) 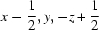.

**Fig. 1.**
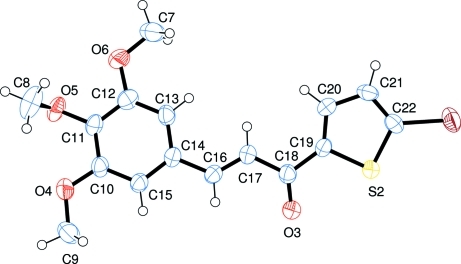
The molecular structure of the title compound. Displacement ellipsoids are drawn at the 50% probability level. The H atoms are shown as spheres of arbitrary radii.

**Fig. 2.**
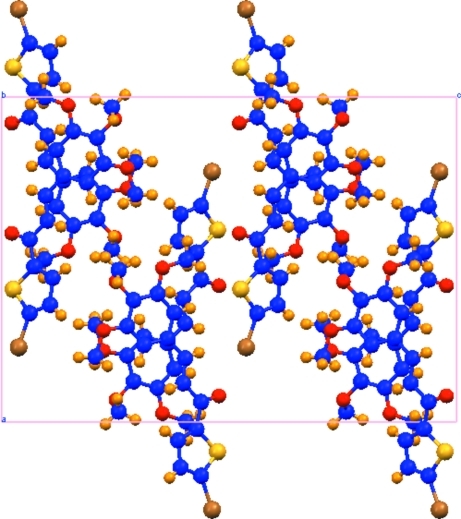
Packing of the molecules when viewed down the b axis.

## supplementary crystallographic information

### Comment

Chalcones are alpha beta unsaturated ketones, widely distributed in nature and are extensively studied for their biological activity (e.g. Chun et al., 2001; Horng et al., 2003; Mei et al., 2003). In this paper we report the crystal structure of the title chalcone derivative, (I) (Fig. 1).

The unit cell contains eight molecules. The five-membered thiophene ring (S2\C19\···C22) is not coplanar with the phenyl ring (C10\C11\···C15) system; the dihedral angle between the two planes is 13.08 (16)°. The crystal structure displays intermolecular C21—H21···O6 and weak intramolecular C8—H8B···O5 and C9—H9B···O4 hydrogen bonds (Table 1). The packing of molecules in the crystal structure is depicted in Fig. 2.

### Experimental

A mixture of 2-acetyl-5-BromoThiophene (0.01 mole) and 3,4,5-trimethoxybenzaldehyde (0.01 mole) were stirred in ethanol (30 ml) and then an aqueous solution of potassium hydroxide (40%,15 ml)was added to it. The mixture was kept over night at room temperature and then it was poured into crushed ice and acidified with dilute hydrochloric acid. The precipiteted chalcone was filtered and crystallized from ethanol to yield colourless prisms of (I).

### Refinement

All H atoms were positioned at calculated positions C—H = 0.93Å for aromatic H and C—H = 0.96Å for methyl H and refined using a riding model with *U*_iso_(H) = 1.2*U*_eq_(C)for aromatic and *U*_iso_(H) = 1.2*U*_eq_(C)for for methyl H.

### Crystal data

**Table:**

C_16_H_15_BrO_4_S	*D*_x_ = 1.578 Mg m^−^^3^
*M**_r_* = 383.25	Melting point: 421 K
Orthorhombic, *P**b**c**a*	Mo *K*α radiation, λ = 0.71073 Å
Hall symbol: -P 2ac 2ab	Cell parameters from 2833 reflections
*a* = 16.8923 (7) Å	θ = 2.4–25.0°
*b* = 8.0793 (6) Å	µ = 2.69 mm^−^^1^
*c* = 23.6427 (17) Å	*T* = 293 K
*V* = 3226.7 (4) Å^3^	Prism, colourless
*Z* = 8	0.22 × 0.15 × 0.12 mm
*F*(000) = 1552	

### Data collection

**Table:**

Oxford Diffraction Xcalibur diffractometer	2833 independent reflections
Radiation source: Mova (Mo) X-ray Source	1944 reflections with *I* > 2σ(*I*)
mirror	*R*_int_ = 0.055
Detector resolution: 16.0839 pixels mm^-1^	θ_max_ = 25.0°, θ_min_ = 2.4°
ω scans	*h* = −20→20
Absorption correction: multi-scan (*CrysAlis PRO RED*; Oxford Diffraction, 2010)	*k* = −9→8
*T*_min_ = 0.625, *T*_max_ = 1.000	*l* = −28→27
17608 measured reflections	

### Refinement

**Table:**

Refinement on *F*^2^	Secondary atom site location: difference Fourier map
Least-squares matrix: full	Hydrogen site location: inferred from neighbouring sites
*R*[*F*^2^ > 2σ(*F*^2^)] = 0.042	H-atom parameters constrained
*wR*(*F*^2^) = 0.074	*w* = 1/[σ^2^(*F*_o_^2^) + (0.0142*P*)^2^] where *P* = (*F*_o_^2^ + 2*F*_c_^2^)/3
*S* = 0.99	(Δ/σ)_max_ = 0.001
2833 reflections	Δρ_max_ = 0.37 e Å^−^^3^
200 parameters	Δρ_min_ = −0.30 e Å^−^^3^
0 restraints	Extinction correction: *SHELXL97* (Sheldrick, 2008), Fc^*^=kFc[1+0.001xFc^2^λ^3^/sin(2θ)]^-1/4^
Primary atom site location: structure-invariant direct methods	Extinction coefficient: 0.00037 (6)

### Special details

**Table:**

Experimental. *CrysAlis PRO*, Oxford Diffraction Ltd., Version 1.171.33.55 (release 05–01–2010 CrysAlis171. NET) Empirical absorption correction using spherical harmonics, implemented in SCALE3 ABSPACK scaling algorithm.IR (KBr) 1653.9, 1597.8, 1071.2, 811.3 cm^-1. 1^H-NMR (300 MHz, CDCl_3_): δ 7.755–7.806 (s, 2 H, Ar–H), 7.114–7.251 (m, 4H, Ar–H and HC=CH), 3.922–3.942 (s, 9 H, OCH_3_).
Geometry. All s.u.'s (except the s.u. in the dihedral angle between two l.s. planes) are estimated using the full covariance matrix. The cell s.u.'s are taken into account individually in the estimation of s.u.'s in distances, angles and torsion angles; correlations between s.u.'s in cell parameters are only used when they are defined by crystal symmetry. An approximate (isotropic) treatment of cell s.u.'s is used for estimating s.u.'s involving l.s. planes.
Refinement. Refinement of *F*^2^ against ALL reflections. The weighted *R*-factor *wR* and goodness of fit *S* are based on *F*^2^, conventional *R*-factors *R* are based on *F*, with *F* set to zero for negative *F*^2^. The threshold expression of *F*^2^ > 2σ(*F*^2^) is used only for calculating *R*-factors(gt) *etc*. and is not relevant to the choice of reflections for refinement. *R*-factors based on *F*^2^ are statistically about twice as large as those based on *F*, and *R*- factors based on ALL data will be even larger.

### Fractional atomic coordinates and isotropic or equivalent isotropic displacement parameters (Å^2^)

**Table:**

	*x*	*y*	*z*	*U*_iso_*/*U*_eq_	
Br1	0.22995 (2)	0.12039 (5)	0.462527 (19)	0.05957 (17)	
S2	0.40847 (5)	0.19214 (12)	0.47450 (4)	0.0457 (3)	
O3	0.58044 (13)	0.2459 (3)	0.47770 (11)	0.0554 (7)	
O4	0.97395 (13)	0.0142 (3)	0.35359 (11)	0.0548 (7)	
O5	0.93207 (14)	−0.0951 (3)	0.25109 (10)	0.0536 (7)	
O6	0.78125 (15)	−0.1434 (3)	0.22387 (10)	0.0584 (8)	
C7	0.7010 (2)	−0.1558 (6)	0.20613 (17)	0.0786 (15)	
H7A	0.6988	−0.2054	0.1693	0.118*	
H7B	0.6779	−0.0473	0.2047	0.118*	
H7C	0.6721	−0.2231	0.2325	0.118*	
C8	0.9680 (2)	−0.2507 (5)	0.25738 (19)	0.0836 (16)	
H8A	1.0071	−0.2655	0.2283	0.125*	
H8B	0.9285	−0.3358	0.2543	0.125*	
H8C	0.9929	−0.2572	0.2938	0.125*	
C9	0.9990 (2)	0.0853 (5)	0.40581 (16)	0.0609 (12)	
H9A	1.0557	0.0825	0.4080	0.091*	
H9B	0.9770	0.0233	0.4367	0.091*	
H9C	0.9811	0.1979	0.4079	0.091*	
C10	0.8948 (2)	0.0080 (4)	0.34261 (15)	0.0413 (9)	
C11	0.8748 (2)	−0.0580 (4)	0.29009 (14)	0.0394 (9)	
C12	0.7953 (2)	−0.0746 (5)	0.27587 (15)	0.0451 (10)	
C13	0.7371 (2)	−0.0232 (4)	0.31290 (15)	0.0433 (10)	
H13	0.6841	−0.0355	0.3031	0.052*	
C14	0.7572 (2)	0.0468 (4)	0.36467 (15)	0.0379 (9)	
C15	0.8364 (2)	0.0618 (4)	0.37962 (14)	0.0420 (9)	
H15	0.8502	0.1078	0.4143	0.050*	
C16	0.69605 (19)	0.1080 (4)	0.40313 (15)	0.0426 (9)	
H16	0.7133	0.1705	0.4338	0.051*	
C17	0.61970 (19)	0.0843 (4)	0.39899 (14)	0.0424 (9)	
H17	0.6011	0.0188	0.3695	0.051*	
C18	0.5618 (2)	0.1556 (4)	0.43846 (15)	0.0402 (9)	
C19	0.47821 (19)	0.1147 (4)	0.42830 (14)	0.0364 (9)	
C20	0.4436 (2)	0.0223 (4)	0.38785 (14)	0.0448 (10)	
H20	0.4718	−0.0289	0.3590	0.054*	
C21	0.3606 (2)	0.0105 (4)	0.39334 (15)	0.0482 (10)	
H21	0.3281	−0.0490	0.3690	0.058*	
C22	0.3348 (2)	0.0965 (4)	0.43839 (15)	0.0418 (9)	

### Atomic displacement parameters (Å^2^)

**Table:**

	*U*^11^	*U*^22^	*U*^33^	*U*^12^	*U*^13^	*U*^23^
Br1	0.0319 (2)	0.0731 (3)	0.0738 (3)	0.0005 (2)	0.0004 (2)	−0.0001 (3)
S2	0.0331 (5)	0.0599 (7)	0.0441 (6)	0.0009 (5)	0.0020 (4)	−0.0137 (5)
O3	0.0377 (14)	0.0760 (19)	0.0525 (17)	0.0036 (14)	−0.0002 (13)	−0.0232 (15)
O4	0.0358 (15)	0.081 (2)	0.0480 (16)	0.0026 (14)	0.0056 (13)	−0.0046 (15)
O5	0.0545 (16)	0.0651 (18)	0.0411 (15)	0.0098 (15)	0.0226 (14)	0.0057 (14)
O6	0.0559 (17)	0.083 (2)	0.0363 (15)	−0.0106 (16)	0.0095 (14)	−0.0114 (15)
C7	0.066 (3)	0.115 (4)	0.055 (3)	−0.029 (3)	0.000 (3)	−0.023 (3)
C8	0.089 (4)	0.075 (4)	0.086 (4)	0.025 (3)	0.039 (3)	0.003 (3)
C9	0.043 (2)	0.078 (3)	0.062 (3)	−0.010 (2)	−0.008 (2)	0.001 (3)
C10	0.035 (2)	0.050 (2)	0.038 (2)	0.0007 (19)	0.0063 (18)	0.0078 (19)
C11	0.042 (2)	0.043 (2)	0.033 (2)	0.0036 (19)	0.0108 (18)	0.0094 (18)
C12	0.054 (2)	0.047 (2)	0.034 (2)	−0.002 (2)	0.006 (2)	0.0072 (19)
C13	0.034 (2)	0.055 (3)	0.041 (2)	−0.0011 (19)	0.0045 (18)	0.004 (2)
C14	0.037 (2)	0.040 (2)	0.037 (2)	0.0061 (18)	0.0056 (18)	0.0036 (18)
C15	0.039 (2)	0.053 (2)	0.034 (2)	0.0055 (19)	0.0024 (18)	0.0007 (19)
C16	0.040 (2)	0.053 (3)	0.034 (2)	0.005 (2)	0.0021 (18)	−0.0043 (19)
C17	0.036 (2)	0.052 (2)	0.039 (2)	−0.0002 (19)	0.0052 (18)	−0.0038 (19)
C18	0.036 (2)	0.049 (2)	0.036 (2)	0.0041 (19)	0.0008 (18)	0.0045 (19)
C19	0.0360 (19)	0.040 (2)	0.0327 (19)	0.0027 (18)	0.0062 (17)	−0.0003 (18)
C20	0.045 (2)	0.054 (3)	0.036 (2)	−0.001 (2)	0.0046 (18)	−0.0100 (19)
C21	0.045 (2)	0.062 (3)	0.037 (2)	−0.011 (2)	−0.0103 (19)	−0.006 (2)
C22	0.0327 (19)	0.047 (2)	0.045 (2)	−0.0008 (18)	−0.0077 (18)	0.003 (2)

### Geometric parameters (Å, °)

**Table:**

Br1—C22	1.871 (3)	C10—C15	1.389 (4)
S2—C22	1.695 (3)	C10—C11	1.393 (5)
S2—C19	1.724 (3)	C11—C12	1.390 (5)
O3—C18	1.221 (4)	C12—C13	1.381 (5)
O4—C10	1.363 (4)	C13—C14	1.390 (5)
O4—C9	1.426 (4)	C13—H13	0.9300
O5—C11	1.370 (4)	C14—C15	1.389 (4)
O5—C8	1.404 (4)	C14—C16	1.462 (4)
O6—C12	1.370 (4)	C15—H15	0.9300
O6—C7	1.423 (4)	C16—C17	1.308 (4)
C7—H7A	0.9600	C16—H16	0.9300
C7—H7B	0.9600	C17—C18	1.469 (4)
C7—H7C	0.9600	C17—H17	0.9300
C8—H8A	0.9600	C18—C19	1.470 (4)
C8—H8B	0.9600	C19—C20	1.347 (4)
C8—H8C	0.9600	C20—C21	1.410 (5)
C9—H9A	0.9600	C20—H20	0.9300
C9—H9B	0.9600	C21—C22	1.344 (5)
C9—H9C	0.9600	C21—H21	0.9300
			
C22—S2—C19	90.97 (17)	C12—C13—C14	120.5 (3)
C10—O4—C9	118.1 (3)	C12—C13—H13	119.7
C11—O5—C8	115.5 (3)	C14—C13—H13	119.7
C12—O6—C7	117.3 (3)	C15—C14—C13	119.6 (3)
O6—C7—H7A	109.5	C15—C14—C16	119.5 (3)
O6—C7—H7B	109.5	C13—C14—C16	120.9 (3)
H7A—C7—H7B	109.5	C14—C15—C10	119.8 (3)
O6—C7—H7C	109.5	C14—C15—H15	120.1
H7A—C7—H7C	109.5	C10—C15—H15	120.1
H7B—C7—H7C	109.5	C17—C16—C14	126.9 (4)
O5—C8—H8A	109.5	C17—C16—H16	116.6
O5—C8—H8B	109.5	C14—C16—H16	116.6
H8A—C8—H8B	109.5	C16—C17—C18	123.4 (3)
O5—C8—H8C	109.5	C16—C17—H17	118.3
H8A—C8—H8C	109.5	C18—C17—H17	118.3
H8B—C8—H8C	109.5	O3—C18—C17	123.1 (3)
O4—C9—H9A	109.5	O3—C18—C19	120.4 (3)
O4—C9—H9B	109.5	C17—C18—C19	116.6 (3)
H9A—C9—H9B	109.5	C20—C19—C18	131.1 (3)
O4—C9—H9C	109.5	C20—C19—S2	110.7 (3)
H9A—C9—H9C	109.5	C18—C19—S2	118.1 (3)
H9B—C9—H9C	109.5	C19—C20—C21	113.8 (3)
O4—C10—C15	124.5 (3)	C19—C20—H20	123.1
O4—C10—C11	115.0 (3)	C21—C20—H20	123.1
C15—C10—C11	120.6 (3)	C22—C21—C20	111.1 (3)
O5—C11—C12	119.9 (3)	C22—C21—H21	124.4
O5—C11—C10	120.8 (3)	C20—C21—H21	124.4
C12—C11—C10	119.2 (3)	C21—C22—S2	113.3 (3)
O6—C12—C13	124.6 (4)	C21—C22—Br1	127.0 (3)
O6—C12—C11	115.1 (3)	S2—C22—Br1	119.6 (2)
C13—C12—C11	120.3 (4)		
			
C9—O4—C10—C15	−2.0 (5)	O4—C10—C15—C14	−178.7 (3)
C9—O4—C10—C11	178.0 (3)	C11—C10—C15—C14	1.3 (5)
C8—O5—C11—C12	−100.4 (4)	C15—C14—C16—C17	−170.3 (4)
C8—O5—C11—C10	84.3 (4)	C13—C14—C16—C17	10.9 (6)
O4—C10—C11—O5	−6.9 (5)	C14—C16—C17—C18	−177.6 (3)
C15—C10—C11—O5	173.1 (3)	C16—C17—C18—O3	1.6 (6)
O4—C10—C11—C12	177.8 (3)	C16—C17—C18—C19	−179.1 (3)
C15—C10—C11—C12	−2.2 (5)	O3—C18—C19—C20	178.5 (4)
C7—O6—C12—C13	2.8 (5)	C17—C18—C19—C20	−0.8 (6)
C7—O6—C12—C11	−177.0 (3)	O3—C18—C19—S2	−2.1 (5)
O5—C11—C12—O6	5.8 (5)	C17—C18—C19—S2	178.6 (2)
C10—C11—C12—O6	−178.8 (3)	C22—S2—C19—C20	0.5 (3)
O5—C11—C12—C13	−174.0 (3)	C22—S2—C19—C18	−179.1 (3)
C10—C11—C12—C13	1.3 (5)	C18—C19—C20—C21	178.9 (3)
O6—C12—C13—C14	−179.4 (3)	S2—C19—C20—C21	−0.5 (4)
C11—C12—C13—C14	0.4 (6)	C19—C20—C21—C22	0.3 (5)
C12—C13—C14—C15	−1.4 (5)	C20—C21—C22—S2	0.1 (4)
C12—C13—C14—C16	177.4 (3)	C20—C21—C22—Br1	179.2 (3)
C13—C14—C15—C10	0.5 (5)	C19—S2—C22—C21	−0.3 (3)
C16—C14—C15—C10	−178.3 (3)	C19—S2—C22—Br1	−179.5 (2)

### Hydrogen-bond geometry (Å, °)

**Table:**

*D*—H···*A*	*D*—H	H···*A*	*D*···*A*	*D*—H···*A*
C21—H21···O6^i^	0.93	2.46	3.320 (4)	155

Symmetry codes: (i) *x*−1/2, *y*, −*z*+1/2.
